# P069. Osmophobia in children with headache

**DOI:** 10.1186/1129-2377-16-S1-A76

**Published:** 2015-09-28

**Authors:** Francesca M Bosetti, Roberta Rossi, Marialia Repici, Corinna Garrone, Barbara Lauria, Emanuele Castagno, Antonella Versace, Giulia Grasso, Rosaura Pagliero

**Affiliations:** Centro Cefalee dell'Età Evolutiva, SC Pediatria d'Urgenza OIRM, AO Città della Salute e della Scienza, Turin, Italy

## Introduction

Osmophobia, or olfactory hypersensitivity, has variable prevalence in children with headache and was proposed as an additional criterion for the diagnosis of migraine in the appendix of the International Classification of Headache Disorders (ICHD)-III beta[1], showing low sensitivity and high specificity for this disturbance[2–4]. The aim of our retrospective study was to define the prevalence of osmophobia in a paediatric population with headache.

## Methods

All the children admitted for headache to a Pediatric Headache Centre from 01/01/2013 to 31/12/2014 were included in the study. For children admitted in 2014, the diagnosis was carried out according to the ICHD-III beta criteria; for those admitted in 2013 the diagnosis was revised according to the same classification. We investigated the presence of osmophobia and type of offending smells reported by the patients.

## Results

We included 482 subjects (259 females): mean age 10.2 years (range 2.5-17.5). Three hundred and forty-four patients (71.3%) had a diagnosis of migraine without aura, 25 migraine with aura (5.1%); 93 children (19.2%) showed tension-type headache; 17 children (3.5%) had an unspecified form of headache or headache not elsewhere classified; 3 patients (0.62%) presented episodic syndromes that may be associated with migraine. Out of 39 patients (8.1%) reporting osmophobia, almost all had migraine (36 without aura, 2 with aura) and only 1 was diagnosed as tension-type headache. Osmophobia was a symptom detected in 10.4% of patients with migraine without aura and in 8% with migraine with aura. The most frequent offending smells were: perfume in 12 cases (30%), 9 food (22.5%), 4 cigarette smoke (10%), 2 food plus perfume (5%), 1 perfume plus cigarette smoke (2.5%), 5 other odours (15%) and 6 undefined (15%).

## Conclusions

Our study showed a lower prevalence of osmophobia in children with headache than that reported in the literature. However, we confirm that osmophobia is more specific for migraine without aura. As already demonstrated in adults and younger patients, osmophobia should be considered in clinical practice as a peculiar symptom useful in the differential diagnosis between migraine without aura and tension-type headache in childhood.

Written informed consent to publication was obtained from the patient(s).

## References

[1] Headache Classification Subcommittee of the International Headache Society (2013). The International Classification of Headache Disorders: 3rd edition (beta version). Cephalalgia.

[2] Corletto E, Dal Zotto L, Resos A, Tripoli E, Zanchin G, Bulfoni C, Battistella PA (2008). Osmophobia in juvenile primary headaches. Cephalalgia.

[3] Zanchin G, Dainese F, Trucco M, Mainardi F, Mampreso E, Maggioni F (2007). Osmophobia in migraine and tension-type headache and its clinical features in patients with migraine. Cephalalgia.

[4] Rocha-Filho PAS, Marques KS, Santos RC Torres, Ribas KN Leal (2015). Osmophobia and headaches in primary care: prevalence, associated factors, and importance in diagnosing migraine. Headache.

